# The Use of Single Therapy With Tocilizumab Versus Combination Therapy With Remdesivir and Tocilizumab in SARS-CoV-2 Patients in El Paso, Texas

**DOI:** 10.7759/cureus.16351

**Published:** 2021-07-13

**Authors:** Nouf K Almaghlouth, Felix E Anyiam, Sidra Shah, Syed Haq, Mohamed J Attia, Roberto Guevara, Suresh Antony

**Affiliations:** 1 Department of Medicine, MountainView Regional Medical Center, Las Cruces, USA; 2 Department of Medicine, Burrell College of Osteopathic Medicine, Las Cruces, USA; 3 Centre for Health and Development (CHD), University of Port Harcourt, Port Harcourt, NGA; 4 Department of Emergency Medicine, Kasr Alainy University Hospitals, Cairo, EGY; 5 Department of Clinical Pharmacy, The Hospitals of Providence Transmountain Campus, El Paso, USA; 6 Department of Medicine, Texas Tech University Health Sciences Center, El Paso, USA

**Keywords:** sars cov-2, tocilizumab, covid-19, remdesivir, clinical outcomes, ventilation, mortality

## Abstract

Background: Currently, the management of SARS-CoV-2 varies with no definitive clinical guidelines, as scientific evidence across the globe differs in therapeutic options. This study intended to provide some clarity to the insufficient data based on the role of monotherapy with tocilizumab (TCZ) and combination therapy with remdesivir (RDV) and TCZ among patients with SARS-CoV-2 infection in El Paso, Texas. We evaluated the use of each therapy in the presence of steroids as the standard of care.

Methods: One hundred and fifty-four SARS-CoV-2-infected patients from four different medical centers in El Paso, Texas, were screened, with 113 eligible for this longitudinal comparative observational study (February 1, 2020 to October 31, 2020). Group 1 (80 patients) received TCZ in the first 24 hours following admission, then methylprednisolone for the next 72 hours and group 2 (33 patients) were given TCZ as detailed in the single therapy group, plus RDV within the first 24 hours. Mann Whitney U test assessed median differences in laboratory biomarkers and Bivariate Logistic Regression assessed the odds of risk. An observation is considered statistically significant when P-value is ≤0.05.

Results: Patients in group 1 had a statistically significant lower odds for ventilation use than group 2 (OR=0.34, 95%CI=0.12-0.95, p=0.034), although no statistically significant difference in mortality outcomes was observed across groups (OR=0.43, 95%CI:0.13-1.39, p=0.269).

Conclusions: We concluded that the use of TCZ in SARS-CoV-2-infected patients in El Paso, with or without RDV, reported no mortality benefit. However, some minimal/non-use of ventilation benefit was observed in group 1. Nonetheless, a randomized controlled trial study is recommended to ultimately determine the combination role of TCZ and RDV among this highly vulnerable group of patients.

## Introduction

In late 2019, a novel coronavirus, known as severe acute respiratory distress syndrome (ARDS) coronavirus 2 (SARS-CoV-2), appeared in Wuhan, central China [1]. The infection with the SARS-CoV-2 causes a respiratory disease that often leads to progressive viral pneumonia and ARDS. Preliminary data illustrated how the host immune response reacts to this type of coronavirus leading to the proliferation of pro-inflammatory cytokines, significant endothelial damage, and generalized vascular complications [2].

Since the recognition of early reported cases of SARS-CoV-2 in December, there have been significant implemented measures to control the spread of the virus and reduce the mortality rates. Yet, despite various pharmacological attempts, the virus remained elusive to any standardized control protocol. The majority of treatment modalities focus primarily on symptomatic relief, prevention of disease progression, and a further reduction in associated mortality and adverse outcomes. Tocilizumab (TCZ), an interleukin-6 (IL-6) inhibitor, was one of the agents used early on for its anti-inflammatory effects, and immunomodulation response in the cytokine release syndrome (CRS) provoked by the virus [3]. Also, remdesivir (RDV), a nucleotide analog prodrug that inhibits viral RNA polymerases, has demonstrated in vitro activity against SARS-CoV-2 [4,5]. Moreover, RDV shortens the time to recovery of SARS-CoV-2 hospitalized individuals with increased oxygen requirement and was thought to have a positive influence on mortality outcomes and a good safety profile [4].

Therefore, different treatment strategies have been proposed in the management of the disease. Nonetheless, the field is still lacking enough evidence-based interventions to reduce mortality and morbidity outcomes. Our study examines the role of single monotherapy with TCZ and combination therapy of RDV and TCZ among patients in El Paso, Texas.

## Materials and methods

Study design

Extraction of literature on SARS-CoV-2 disease and mortality that guided the review for the present study were conducted using PubMed, Google Scholar, and Medline. The study design was a longitudinal observational study within El Paso, Texas hospitals from February 1, 2020 to October 31, 2020, of 154 patients positive with SARS-CoV-2 PCR, oxygen supplementation (>3 L), Pneumonia Severity Index (PSI) scores 130 or less and been 18 years and more. SARS-CoV-2 PCR negative patients and those experiencing reactions/hypersensitivity to either TCZ/RDV, and whose lungs have been compromised either via active TB infection, ALT or AST, or other comorbid conditions like cancer, renal disease, or cardiac arrhythmia were excluded. During study commencement, patients received TCZ-only, and the other group TCZ and RDV.

Procedures

Patients in group 1, in the first 24 hours and 72 hours of hospitalization, received 4 mg/kg per day of TCZ - q12hr - and 60 mg of methylprednisolone - q8hr. Patients in group 2, in the first 24 hours and 72 hours of hospitalization, received 4 mg/kg per day of TCZ - q12hr, plus RDV, and 60 mg of methylprednisolone - q8hr. In the event of an anticipated secondary bacterial infection, ceftriaxone and azithromycin are added. On admission, radiological imaging, and laboratory biomarkers were assessed on days 3 and 6, for the following parameters: complete blood count (CBC), complete metabolic panel (CMP), C-reactive protein (CRP), lactate dehydrogenase (LDH), ferritin, D-dimer, IL-6, and procalcitonin. All these laboratory markers were monitored throughout the stay. The length of hospital stay was monitored for each patient.

Statistical analysis

Data were extracted from patients’ folders into Microsoft Excel ® version 2019 where it was coded and cleaned and then imported into Stata/IC 16.0 (StataCorp, College Station, TX) for statistical analysis. Categorical data from the explanatory variables such as the socio-demographic and clinical characteristics were presented in the form of percentages (%) and frequencies with results presented in tables, while continuous data from laboratory parameters were presented in median and IQR after the Shapiro-Wilk normality test shows the data distribution was non-normal. Non-parametric data analysis using the Mann-Whitney U test was utilized for associating specific continuous independent variables with clinical outcomes. Bivariate Logistic Regression analysis was conducted to examine the odds of risk association (represented by odds ratio, ORs). All ORs were outlined with their 95% CI and relative p-values. An observation is considered statistically significant if the “p-value is less than or equal to 0.05 (≤0.05)” at a 95% confidence interval.

## Results

Totally, 113/154 SARS-CoV-2-infected patients were included in this study based on the inclusion and exclusion criteria. The group of single therapy with TCZ (group 1) consisted of 80 patients and the second group which was treated with combination therapy of TCZ and RDV (group 2) consisted of 33 patients. Both groups received steroids as the standard of care. The summary of sociodemographic characteristics and clinical findings of SARS-CoV-2-infected patients treated with TCZ alone in group 1 as compared to those treated with TCZ plus RDV in group 2 were illustrated in Table 1. Socio-demographic characteristics and clinical findings were equally represented in both groups with no statistical significance observed (p>0.05).

**Table 1:** The association of socio-demographic characteristics, medical history, and clinical presentations among SARS-CoV-2 patients in treatment groups using the Bivariate Logistic Regression (OR) **Statistically significant (p≤0.05). ^n1^Represents the sample size for the Tocilizumab (TCZ) only group (group 1); the sample size of this group varies by variables.

Variables	Treatment groups “both received methylprednisolone”		OR (95% CI)	P-value
	TCZ only group (group 1) n=80	TCZ plus RDV group (group 2) n=33		
Age (n^1^=79)	79	33		
<30	3 (3.80%)	1 (3.03%)	-	-
30–49	16 (20.25%)	10 (30.30%)	1.04 (0.09–11.52)	0.972
50–69	37 (46.84%)	14 (42.42%)	0.56 (0.18–1.72)	0.308
≥70	23 (29.11%)	8 (24.24%)	0.91 (0.33–2.53)	0.871
Gender				
Male	45 (56.96%)	15 (45.45%)	1.58 (0.70–3.64)	0.266
Female	34 (43.04%)	18 (54.55%)		
Diabetes mellitus (n^1^=72)				
Yes	37 (51.39%)	16 (48.48%)	1.12 (0.49–2.59)	0.782
No	35 (48.61%)	17 (51.52%)		
Essential hypertension (n^1^=72)				
Yes	47 (65.28%)	20 (60.61%)	1.22 (0.51–2.87)	0.644
No	25 (34.72%)	13 (39.39%)		
Hyperlipidemia (n^1^=72)				
Yes	18 (25.00%)	7 (21.21%)	1.24 (046–3.53)	0.672
No	54 (75.00%)	26 (78.79%)		
Comorbidities (n^1^=72)				
>2	26 (36.11%)	15 (45.45%)	0.68 (0.29–1.59)	0.362
≤2	46 (63.89%)	18 (54.55%)		
Travel history (n^1^=70)				
Yes	11 (15.71%)	1 (3.03%)	5.89 (0.94–133.36)	0.061
No	59 (84.29%)	32 (96.97%)		
Contact history (n^1^=70)				
Yes	34 (48.57%)	9 (27.27%)	2.50 (1.02–6.40)	0.067
No	36 (51.43%)	24 (72.73%)		
Fever (n^1^=72)				
Yes	53 (73.61%)	18 (54.55%)	2.30 (0.96–5.54)	0.086
No	19 (26.39%)	15 (45.45%)		
Cough (n^1^=72)				
Yes	49 (68.06%)	22 (66.67%)	1.06 (0.43–2.56)	0.888
No	23 (31.94%)	11 (33.33%)		
Shortness of breath (n^1^=72)				
Yes	62 (86.11%)	29 (87.88%)	0.86 (0.22–2.91)	0.805
No	10 (13.89%)	4 (12.12%)		
Other symptoms (n^1^=72)				
Yes	42 (58.33%)	24 (72.73%)	0.53 (0.21–1.29)	0.156
No	30 (41.67%)	9 (27.27%)		
Symptoms (n^1^=72)				
>2	52 (72.22%)	24 (72.73%)	0.98 (0.37–2.45)	0.957
≤2	20 (27.78%)	9 (27.27%)		
Bacterial coinfections				
Yes	12 (15.00%)	5 (15.15%)	0.99 (0.32–3.38)	0.984
No	68 (85.00%)	28 (84.85%)		
Multi-organ damage				
Yes	10 (12.50%)	0 (0.00%)	0.00 (0.00–1.26)	0.999
No	70 (87.50%)	33 (100.00%)		

After the application of the Mann-Whitney U test, we noted statistically significant results between average IL-6, white blood cell count (WBC) levels with treatment groups. A statistically significant increased median IL-6 values were noted among those given only TCZ compared to those that received TCZ plus RDV (511.33 vs. 199.0) with a P-value (0.007). For WBC values, a statistically significant increased median WBC values were noted among those given the combination therapy compared to those given TCZ-only therapy (9.90 vs. 7.55) with a P-value of 0.003. However, no statistically significant relationship between treatment groups was observed among CRP, procalcitonin, ferritin, LDH, D-dimer, hemoglobin (Hgb), hematocrit (HCT), and partial thromboplastin time (PTT), as illustrated in Table 2.

**Table 2:** Laboratory biomarkers and treatment groups (Mann Whitney U test) *Note; IL-6, normal 0.0–12.2 (pg/mL); CRP, normal <8.0 (mg/L); procalcitonin, normal 0.10–0.49 (ng/mL); ferritin, normal 12–300 for males, 12–150 for females (ng/mL); LDH, normal 109–245 (U/L); D-dimer, normal <0.5 (mcg/mL). **Statistically significant (p≤0.05). TCZ: tocilizumab: RDV: remdesivir, IL-6: interleukin-6, CRP: C-reactive protein, LDH: lactate dehydrogenase, WBC: white blood cell, Hgb: hemoglobin, HCT: hematocrit, PTT: partial thromboplastin time.

Lab biomarkers	Treatment groups “both received methylprednisolone”		Z test	p-Value
	TCZ only group (group 1) median (IQR)	TCZ plus RDV group (group 2) median (IQR)		
Average IL-6	511.33 (275.03–703.08)	199.0 (34.25–402.20)	7.39	0.007**
Average CRP	6.31 (4.19–11.55)	9.45 (4.81–14.52)	2.33	0.127
Average ferritin	571.56 (355.55–936.52)	494.0 (184.75–761.75)	3.29	0.070
Average procalcitonin	0.47 (0.33–0.70)	0.70 (0.35–7.10)	0.86	0.354
Average LDH	371.30 (290.67–459.67)	338.0 (283.0–420.50)	0.54	0.464
Average D-dimer	1.13 (0.69–1.97)	0.96 (0.69–6.85)	0.73	0.393
Average WBC count	7.55 (5.96–9.43)	9.90 (8.13–11.45)	13.24	0.0003**
Average Hgb	13.40 (12.20–14.33)	13.30 (12.25–14.70)	0.11	0.744
Average HCT	40.70 (36.20–43.27)	39.43 (26.73–44.40)	0.09	0.765
Average PTT	31.90 (21.34–36.70)	30.20 (28.03–39.0)	0.32	0.573

Patients in group 1 have statistically significantly lower odds for ventilation use compared to patients in group 2 (OR=0.34, 95%CI=0.12-0.95, p=0.034). On the other hand, there was no statistically significant difference between groups 1 and 2 in mortality outcomes (OR=0.43, 95%CI:0.13-1.39, p=0.269), as shown in Table 3.

**Table 3:** Clinical outcomes and treatment groups using the Bivariate Logistic regression (OR) **Statistically significant (p ≤ 0.05).

Outcomes	Treatment groups “both received methylprednisolone”		OR (95% CI)	P-value
	TCZ only group (group 1)	TCZ plus RDV group (group 2)		
Ventilation				
Ventilated	9 (11.25%)	9 (27.27%)	0.34 (0.12–0.95)	0.034**
Non-ventilated	71 (88.75%)	24 (72.73%)		
Mortality				
Deceased	7 (8.75%)	6 (18.18%)	0.43 (0.13–1.39)	0.269
Non-deceased	73 (91.25%)	27 (81.82%)		

## Discussion

The population of this study is unique as it reflects a primarily Hispanic demographic population in El Paso with discrete genetics, background characteristics, and higher predisposition to diabetes, and obesity than the rest of the United States (US). Taking into consideration that the city of El Paso was one of the cities in the US experiencing extraordinarily higher rates of outbreak since the beginning of the pandemic [6]. El Paso County was considered at an extremely high-risk level, specifically with the peak of cases in the month of November. The number of new cases in the County reached up to 3,538 on November 4 [6]. It was estimated that at least one in seven residents have been infected in the County since February of the same year, and a total of 117,743 reported cases [6]. Therefore, this study reflects data on the highly vulnerable population affected by SARS-CoV-2 in Texas.

We inferred that the use of TCZ in SARS-CoV-2-infected patients in El Paso, with or without RDV, reported no mortality benefit. TCZ-only treated group showed a lower odd for ventilation use with only (11.25%) as compared to patients who received the combination therapy (27.27%).

In a previous pilot study conducted by Antony et al. [7], it concluded that early administration of TCZ followed by methylprednisolone might reduce the demand for mechanical ventilation and decrease certain inflammatory markers including CRP, ferritin, LDH, and D-dimer levels. It is the same findings we observed in both of our treatment groups. However, those who received TCZ and RDV were noted to have a statistically significantly higher proportion for ventilation use. This can be justified by the level of severity index of the illness in the combination group being higher than those receiving the single therapy with TCZ. Also, the time of onset of symptoms is thought to be slightly longer before initiation of TCZ in group 2, as opposed to group 1. Therefore, the initiation of TCZ and its efficacy is primarily time-sensitive.

In a study conducted by Xu et al. [8], clinical improvements were observed in the use of TCZ, especially in respiratory function, which was shown to lower oxygen requirement and minimize lung opacity on radiological imaging [8].

Furthermore, findings from the CORIMUNO-19 [9] and EMPACTA (Evaluating Minority Patients with Actemra) [10] studies also showed that TCZ caused a reduction in the need for mechanical ventilation, which on the other hand, improved ICU-level care patients. Interestingly, a randomized controlled trial (RCT) newly published by the RECOVERY group [11] has concluded that patients receiving TCZ were more likely to be discharged alive from a medical facility within a 28-day period. Additionally, the latter study also noted that patients who received TCZ and were not on mechanical ventilation at baseline were less likely to have mortality or use invasive mechanical ventilation as compared to those receiving steroids alone.

A recent RCT study has shown no benefits of TCZ or in combination with, RDV in the prevention of mortality or mechanical ventilation in those patients moderately affected by SARS-CoV-2 infection [12].

Moreover, in a WHO Solidarity Trial Consortium, whose primary outcome was to evaluate effects on in-hospital mortality among SARS-CoV-2 patients, and secondary outcomes were initiation of ventilation and duration of hospitalizations, RDV failed to show benefit in mortality, initiation of ventilation, or the length of hospitalization [13]. Another study showed high mortality despite the use of RDV [14].

Similar findings had also observed no significant differences in either group in clinical and laboratory characteristics, which is in line with findings from the present study De Rosa et al. [15]. Although in contrast to our study, Pasquini et al. [16] observed treatment benefits relating to the survival of patients with TCZ in combination with RDV [16].

One report [17], recently published by Akinosoglou et al. using a combination of TCZ and RDV, observed that using RDV should be early before secondary uncontrolled pro-inflammatory response initiated. Otherwise, in the presence of a pro-inflammatory reaction, immunomodulation as TCZ can be added. The authors have emphasized that reduction in clinical outcomes should be achieved by combination therapies not particularly limited to TCZ and RDV.

At least one RCT that used TCZ and RDV in SARS-CoV-2 is currently ongoing. Such a study will better define TCZ and RDV's role as a combination in the management of this infection [18].

A number of limitations were observed. First, due to the study’s observational design, confounders' impact is not completely ruled out especially in the presence of comorbidities that may affect the clinical outcomes. Second, the number of eligible participants included. In addition, this study was conducted in a group of hospitals representing a single center and one location that may not be representative enough. However, with the significant impact caused by SARS-CoV-2, examining the combination of TCZ and RDV in a longitudinal comparative observational study is considered the first of its kind.

## Conclusions

We concluded that the use of TCZ in SARS-CoV-2-infected patients in El Paso, with or without RDV, reported no mortality benefit if used late in the disease process. TCZ-only treated group showed a lower odds for ventilation use compared to patients who received the combination. Most importantly, our study design is considered the first of its kind using TCZ and RDV in a longitudinal comparative observational study.

## Appendices

Author contributions

Nouf Almaghlouth led the project, reviewed the literature, performed the statistical analysis, and wrote the final manuscript. Felix Anyiam contributed to data interpretation, revising the performed statistical analysis, and assisted in the writing and editing of the final manuscript. Sidra Shah, Syed Haq, and Mohamed Attia reviewed the literature, computed the data, and helped in writing the final paper draft. Roberto Guevarra contributed to the collection of data and revised this study. Suresh Antony conceptualized the study's primary objectives and contributed to the study design, data acquisition, and the final manuscript review and editing. Suresh Antony and Roberto Guevarra were involved in the care of patients. Nouf Almaghlouth and Felix Anyiam checked all data.

## References

[1] Zhu N, Zhang D, Wang W (2020). A novel coronavirus from patients with pneumonia in China, 2019. N Engl J Med.

[2] Kipshidze N, Dangas G, White CJ (2020). Viral coagulopathy in patients with COVID-19: treatment and care. Clin Appl Thromb Hemost.

[3] Luo P, Liu Y, Qiu L, Liu X, Liu D, Li J (2020). Tocilizumab treatment in COVID-19: a single center experience. J Med Virol.

[4] Malin JJ, Suárez I, Priesner V, Fätkenheuer G, Rybniker J (2020). Remdesivir against COVID-19 and other viral diseases. Clin Microbiol Rev.

[5] Sörgel F, Malin JJ, Hagmann H (2021). Pharmacokinetics of remdesivir in a COVID-19 patient with end-stage renal disease on intermittent haemodialysis. J Antimicrob Chemother.

[6] El Paso County (2021). Tracking Coronavirus in El Paso County, Texas. https://www.nytimes.com/interactive/2021/us/el-paso-texas-covid-cases.html.

[7] Antony SJ, Davis MA, Davis MG (2021). Early use of tocilizumab in the prevention of adult respiratory failure in SARS-CoV-2 infections and the utilization of interleukin-6 levels in the management. J Med Virol.

[8] Xu X, Han M, Li T (2020). Effective treatment of severe COVID-19 patients with tocilizumab. Proc Natl Acad Sci U S A.

[9] Hermine O, Mariette X, Tharaux PL, Resche-Rigon M, Porcher R, Ravaud P (2021). Effect of tocilizumab vs usual care in adults hospitalized with COVID-19 and moderate or severe pneumonia: a randomized clinical trial. JAMA Intern Med.

[10] (2021). Roche - Roche’s phase III EMPACTA study showed Actemra/RoActemra reduced the likelihood of needing mechanical ventilation in hospitalised patients with COVID-19 associated pneumonia. https://www.roche.com/media/releases/med-cor-2020-09-18.htm.

[11] Landray M (2021). Tocilizumab in patients admitted to hospital with COVID-19 (RECOVERY): preliminary results of a randomised, controlled, open-label, platform trial. Lancet.

[12] Stone JH, Frigault MJ, Serling-Boyd NJ (2020). Efficacy of tocilizumab in patients hospitalized with COVID-19. N Engl J Med.

[13] Pan H, Peto R, Henao-Restrepo AM (2021). Repurposed antiviral drugs for COVID-19 -interim WHO SOLIDARITY trial results. N Engl J Med.

[14] Beigel JH, Tomashek KM, Dodd LE (2020). Remdesivir for the treatment of covid-19 - final report. N Engl J Med.

[15] De Rosa RC, Mascolo S, Romanelli A (2020). Remdesivir for the treatment of SARS-CoV-2 infection in patients admitted in ICU: a case series. J Pharmacol Pharm Pharmacovigil.

[16] Pasquini Z, Montalti R, Temperoni C (2020). Effectiveness of remdesivir in patients with COVID-19 under mechanical ventilation in an Italian ICU. J Antimicrob Chemother.

[17] Akinosoglou K, Velissaris D, Ziazias D (2021). Remdesivir and tocilizumab: mix or match. J Med Virol.

[18] (2021). A study to evaluate the efficacy and safety of remdesivir plus tocilizumab compared with remdesivir plus placebo in hospitalized participants with severe COVID-19 pneumonia. https://clinicaltrials.gov/ct2/show/NCT04409262.

